# ASO Author Reflections: Variation in Cost Centers Following Gastrointestinal Cancer Surgery

**DOI:** 10.1245/s10434-025-16934-5

**Published:** 2025-01-21

**Authors:** Eshetu Worku, Mujtaba Khalil, Timothy M. Pawlik

**Affiliations:** Department of Surgery, The Urban Meyer III and Shelley Meyer Chair for Cancer Research, The Ohio State University, Wexner Medical Center and James Comprehensive Cancer Center, Columbus, OH USA

## Past

Cancer diagnosis and treatment impose a significant financial burden on the US healthcare system.^1^ Hospitals strive to deliver high-quality care within various payment systems, including private payers and the Centers for Medicare and Medicaid Services (CMS) Diagnosis-Related Group (DRG) framework.^2^ The CMS DRG-based payment system standardizes payments for comparable medical episodes, aiming to reduce waste and improve efficiency.^2^ Moreover, CMS DRG reimburses hospitals a fixed amount for patient care based on diagnoses and procedures, rather than actual costs incurred.^2^ Notably, significant variations in surgical costs persist within and between hospitals, influenced by hospital type, location, surgical techniques, patient demographics, and comorbidities.^3,4^ Surgical care, a major cost center in US healthcare, has been associated with rising costs and severe health implications for gastrointestinal (GI) cancer patients.^5^ Despite the application of DRG codes in GI cancer care, the ability of the DRG system to reduce cost variation at cost centers is not well understood. Therefore, the current study sought to identify and quantify the variation in cost at hospital cost centers following GI cancer surgery.^6^

## Present

Among 35,908 individuals who underwent pancreatectomy (8.2%), colectomy (79.4%), and proctectomy (12.4%), median age was 78 years (interquartile range 72–84) and approximately one-half of the cohort was male (56.1%). Median Medicare payments varied by cancer type: colectomy ($21,704), pancreatectomy ($26,709), and proctectomy ($21,228) [all *p* < 0.001]. Operating room costs ($6891, 23.82%), hospital stay ($5931, 20.9%), and professional fees ($4352, 15.35%) comprised 60% of total costs. Surgeons had the highest charges (pancreatectomy: $2037; colectomy: $2131; proctectomy: $2243), followed by anesthesiologists (pancreatectomy: $622; colectomy: $431; proctectomy: $480). Total hospital charge variability was primarily influenced by patient factors (83%) followed by surgeon factors (9%) and hospital factors (8%). Comorbidities increased surgical costs by 4.57%, cancer stage by 3.25%, and a low social vulnerability index by 2.75%.

## Future

DRG payment policies allow hospitals within the DRG framework to potentially profit if they treat patients within payment limits, however exceeding these limits can result in financial losses. The DRG model is supposed to encourage efficiency and cost-cutting; however, the DRG model can have drawbacks, including cost-shifting and disparities in care quality, especially at the cost-center level. Hospitals may prioritize less complex cases or select patients with fewer comorbidities, potentially compromising care and not providing some services. In turn, centers with a higher proportion of complex cases and uninsured patients may struggle under the DRG system, facing financial challenges that hinder care quality. As such, DRG payment policies may need to be reviewed in light of costs at the cost-center level to effectively account for patient complexity and hospital resource needs, and enhance value-based care focusing on patient outcomes over cost efficiency. The variation in costs at the cost-center level demonstrated in the current study highlight the importance of reinforcing surgical protocols and standardizing procedures to improve care quality and cost effectiveness.
